# Thin Films of Tungsten Disulfide Grown by Sulfurization of Sputtered Metal for Ultra-Low Detection of Nitrogen Dioxide Gas

**DOI:** 10.3390/nano15080594

**Published:** 2025-04-12

**Authors:** Anastasiya D. Fedorenko, Svetlana A. Lavrukhina, Victor A. Alekseev, Vitalii I. Sysoev, Veronica S. Sulyaeva, Alexander V. Okotrub, Lyubov G. Bulusheva

**Affiliations:** Nikolaev Institute of Inorganic Chemistry SB RAS, 3 Acad. Lavrentiev ave., 630090 Novosibirsk, Russia; fedorenko@niic.nsc.ru (A.D.F.); x-rayspectroscopy@mail.ru (S.A.L.); alekseev@niic.nsc.ru (V.A.A.); sysoeev@niic.nsc.ru (V.I.S.); veronica@niic.nsc.ru (V.S.S.); spectrum@niic.nsc.ru (A.V.O.)

**Keywords:** WS_2_ film, magnetron sputtering, CVD synthesis, chemiresistive sensor, NO_2_, DFT calculations

## Abstract

Tungsten disulfide (WS_2_) is attractive for the development of chemiresistive sensors due to its favorable band gap, as well as its mechanical strength and chemical stability. In this work, we elaborate a procedure for the synthesis of thin films consisting of vertically and/or horizontally oriented WS_2_ nanoparticles by sulfurizing nanometer-thick tungsten layers deposited on oxidized silicon substrates using magnetron sputtering. According to X-ray photoelectron spectroscopy and Raman scattering data, WS_2_ films grown in an H_2_-containing atmosphere at 1000 °C are almost free of tungsten oxide. The WS_2_ film’s thickness is controlled by varying the tungsten sputtering duration from 10 to 90 s. The highest response to nitrogen dioxide (NO_2_) at room temperature was demonstrated by the film obtained using a tungsten layer sputtered for 30 s. The increased sensitivity is attributed to the high surface-to-volume ratio provided by the horizontal and vertical orientation of the small WS_2_ nanoparticles. Based on density functional calculations, we conclude that the small in-plane size of WS_2_ provides many high-energy sites for NO_2_ adsorption, which leads to greater charge transfer in the sensor. The detection limit of NO_2_ calculated for the best sensor (WS_2_-30s) is 15 ppb at room temperature and 8 ppb at 125 °C. The sensor can operate in a humid environment and is significantly less sensitive to NH_3_ and a mixture of H_2_, CO, and CO_2_ gases.

## 1. Introduction

Tungsten disulfide (WS_2_) belongs to a family of two-dimensional (2D) layered compounds that are currently attracting much attention, due among other things to the significant dependency of their properties on the number of layers, in-plane size, and shape of nanoparticles [1,2]. Thus, the band gap of WS_2_ varies from ~1.4 eV for a hexagonal bulk 2H crystal to ~2.1 eV for a hexagonal monolayer [3]. 2D semiconductors with a high surface-to-volume ratio and stability in different chemical environments are promising for sensor applications [4,5]. The WS_2_ layer consists of a plane of tungsten atoms sandwiched between two planes of sulfur atoms, which creates various sites for the adsorption of molecules. The covalent bonding of each W atom to six sulfur neighbors provides a mechanically and chemically robust structure whose electrical conductivity is very sensitive to charge transfer [6,7]. Due to these properties, WS_2_ nanomaterials have the potential to be used in chemiresistive sensors [8,9].

Nitrogen dioxide (NO_2_) is a major air pollutant produced by industrial activities and vehicle emissions [10]. To protect the environment and human health, various materials capable of detecting this gas are being studied. Among them are nanostructured metal oxides, carbon nanomaterials, layered metal-containing compounds, and combinations of these materials [11,12,13]. Recently published examples of NO_2_ sensors made from materials belonging to these classes are collected in Table 1. Metal oxides currently used in commercial gas sensors are very sensitive to NO_2_. However, they typically operate at elevated temperatures. Carbon nanomaterials, particularly single-walled carbon nanotubes (SWCNTs) and reduced graphene oxide (rGO), can detect less than 40 ppb of NO_2_ in air, but the optimal operation temperature is often 100–150 °C. Lower power consuming sensors can be fabricated using 2D materials such as MXenes and transition metal dichalcogenides. Sensor sensitivity and response/recovery are improved by combining different materials. The Occupational Safety and Health Administration has established that exposure to 1 ppm NO_2_ should not exceed 15 min [14], while the exposure limit recommended by the United States Environmental Protection Agency is 53 ppb NO_2_ [15,16]. The change in resistance upon adsorption of the analyte determines the response of the sensor, and in this sense WS_2_, which has a high mobility of charge carries [17], is an attractive material for sensing layers. The data presented in Table 1 on the limit of detection (LOD), response and recovery time, and selectivity to NO_2_ as compared to other air molecules show that WS_2_-containing materials are competitive and can be used in practice. However, the issues of the interactions of WS_2_ with analytes, and especially the role of the basal surface and edges of the layers, require more detailed study to develop advanced sensors.

The first chemiresistive WS_2_ sensors were presented in 2014 [28]. Films on SiO_2_/Si substrates were obtained by plasma-assisted sulfurization of sputtered tungsten oxide WO_3_ and showed high sensitivity to NH_3_ gas at room temperature. Subsequently, WS_2_ sensors that were selective for NO_2_ gas [29], H_2_S gas [30], acetone vapor [31], able to recognize NH_3_ and NO_2_ [32], and demonstrated cross-selectivity for NH_3_ and H_2_S [33] were reported. According to density functional theory (DFT) calculations, the hexagonal WS_2_ monolayer interacts most strongly with the NO_2_ molecule [34,35]. Since the sensor selectivity is primarily determined by the adsorption energy of the analyte, an ideal, thermodynamically stable WS_2_ should preferentially detect NO_2_. Indeed, WS_2_ bilayers extracted from the bulk 2H compound were completely selective for NO_2_ at room temperature [36]. The structural and compositional features of the WS_2_-based sensing material and the working conditions, in particularly atmosphere and temperature, may explain the high sensitivity to other analytes mentioned above.

The key parameters of WS_2_ sensors depend on the number of layers, morphology, defects, and the presence of oxygen. For example, a sensor made of four-layered WS_2_ synthesized through the atomic layer deposition of WO_3_ followed by sulfurization at 1000 °C showed a higher response to NO_2_ and acetone than thinner sensors [31]. However, a sensor prepared from exfoliated WS_2_ bilayers was more sensitive to 1–3 ppm NO_2_ compared to sensors consisting of five and ten layers [36]. Using NH_3_ as an example, it was shown that the recovery rate of the WS_2_ sensor increases with a decrease in the number of nanosheets [37]. The influence of morphology on the sensor properties was clearly demonstrated by comparing WS_2_ triangles and WS_2_ flakes grown on 1D WS_2_ nanostructures through the interaction of sulfur vapor with WO_3_ nanorods and WO_3_ nanoneedles, respectively [38]. At an operation temperature of 150 °C, the triangles exhibited a higher response to NO_2_, while the flakes were better suited for NH_3_ detection. Replacing part of the sulfur with oxygen in WS_2_ flowers prepared using a hydrothermal procedure resulted in a higher and faster response of the sensor to NO_2_ [39]. In contrast, the sensor obtained by drop-casting of a dispersion of WS_2_ flakes followed by annealing in air turned out to be more sensitive to the reducing gases NH_3_ and H_2_ than to the oxidizing gas NO_2_ [40].

An analysis of the works devoted to WS_2_ chemiresistive sensors shows that a sensor’s performance can be a result of the combined action of various structural factors. Reducing the number of adjacent WS_2_ layers allows the surface area for adsorbates to be increased. The overlapping of layers on the substrate is necessary to obtain an electrical signal from the sensor. Thus, it was demonstrated that WS_2_ deposited on a conductive carbon fiber changes conductivity more strongly upon analyte adsorption than its unsupported analogue [15]. At the same time, from a theoretical point of view, the edges of WS_2_ are more reactive toward various gases than the basal plane [41], and experiments confirm this [42,43,44]. Careful study of each structural factor affecting the performance of a 2D layered sensor is important for the targeted creation of effective architectures and combinations with other compounds.

In this work, we synthesized a set of WS_2_ films on SiO_2_/Si substrates and tested the obtained samples as resistive sensors for NO_2_ detection. To prepare the films, tungsten layers of different thickness were deposited on the substrates using magnetron sputtering and then reacted with sulfur vapor mixed with H_2_ at 1000 °C. Previous studies have shown that the thickness of the tungsten seed layer determines the morphology of the WS_2_ film, motivating the growth of layers either parallel or perpendicular to the substrate during sulfurization [45,46]. Different layer orientations affect the electrical conductivity [45] and optical properties [46] of the film. Nanostructured WS_2_ films with high surface-to-volume ratios have shown promise for use in ultraviolet and visible photodetectors [47]. To the best of the authors’ knowledge, such WS_2_ films have not been tested as chemiresistive sensors. Here we fill this gap. A set of WS_2_ films was synthesized under the same sulfurization conditions using W seed layers sputtered for 20–90 s and characterized by scanning electron microscopy (SEM), Raman scattering, and X-ray photoelectron spectroscopy (XPS). The best WS_2_-30s sensor was able to detect ultra-low concentrations of NO_2_ in air at room temperature, though it lacked a fast recovery. However, the recovery time improved significantly at 125 °C. The synthesis method used here has the advantage of producing large-area WS_2_ films with controlled thickness and morphology. In addition, it is inexpensive and scalable. The WS_2_ films can be grown on various desired substrates and used to deposit different compounds on their surface to enhance and optimize the sensor’s performance.

## 2. Materials and Methods

### 2.1. Materials Synthesis and Characterization

Substrates cut from a single-crystal silicon wafer were annealed in air at 1050 °C for 16 h to form a surface oxidized layer ~300 nm thick. The substrates were thoroughly cleaned using hot mineral acids and placed in a magnetron sputtering system (OJSC Vacuum Systems, Novosibirsk, Russia). The system was evacuated to a pressure of ~10^−4^ mbar and the SiO_2_/Si substrates were annealed at a temperature of 250 °C for 30 min, then tungsten was deposited in an argon atmosphere (~5.4 × 10^−3^ mbar) at a power of 150 W for tens of seconds. A two-zone horizontal quartz chemical vapor deposition (CVD) reactor was used to synthesize WS_2_ films. The substrates were placed in a high-temperature Zone I and a boat with elemental S was placed in a low-temperature Zone II. The reactor was evacuated to ~10^−2^ mbar by a fore vacuum pump 2NVR-5DM (JSC “Vacuummash”, Kazan, Russia) and purged with an Ar flow at a rate of 150 sccm. Zone I was then heated to 150 °C to anneal the substrates for 30 min. Then Zone I was heated to 1000 °C and Zone II was heated to 200 °C. Sulfur vapor was transferred from Zone II to Zone I in a flow of argon (12 sccm) and H_2_ (1.2 sccm) at atmospheric pressure. The reaction was carried out for 60 min, then the furnaces were switched off and the samples were cooled naturally in an Ar flow of 150 sccm. The resulting films are designated as WS_2_-10s, WS_2_-20s, WS_2_-30s, WS_2_-40s, WS_2_-50s, and WS_2_-90s depending on the deposition time of the W layer.

The structure of the samples was studied by SEM and Raman spectroscopy. SEM images were obtained using a CIQTEK SEM5000 (CIQTEK, Ltd., Hefei, China) at an accelerating voltage of 15 kV. Raman scattering was excited by an Ar^+^ laser with a wavelength of 514 nm using a LabRAM HR Evolution spectrometer (Horiba, Ltd., Kyoto, Japan). The composition of the samples was determined using the XPS method. Measurements were performed on a FlexPS spectrometer (SPECS GmbH, Berlin, Germany) at room temperature using monochromatic Al Kα (1486.71 eV) radiation. The surface concentration of the elements was determined from the XPS survey spectra, taking into account the photoelectron cross-sections. The analysis of fine lines involved Shirley background subtraction and curve fitting using a Gaussian/Lorenzian product function in CasaXPS software, version 2.3.24 (Casa Software, Ltd., Teignmouth, UK). Binding energies were calibrated using the C 1s line at 284.6 eV originating from adventitious surface carbon.

### 2.2. Sensors Fabrication and Tests

WS_2_ films on SiO_2_/Si substrates were tested using the laboratory setup schematically presented in Figure S1. To fabricate the sensor element, two silver contacts were deposited on the sample surface at a distance of ~3 mm and connected to the measuring cell using a gold wire with a diameter of 50 μm. The electrical signal was monitored with a Keithley 6485 picoammeter (Keithley Instruments, Inc., Cleveland, OH, USA) at a constant voltage in the range of 0.5–4 V depending on the sample’s resistance. To achieve a steady state, each element was stabilized by heating in an argon atmosphere at a temperature of 150 °C for 40 min, followed by natural cooling in this atmosphere to room temperature, and then exposure to dry air (H_2_O content < 1 ppm) for 30 min. The treatment temperature was chosen based on the photoluminescence study of the WS_2_ monolayer, which did not reveal a change in the charge state of the sample when heated to 150 °C [48].

The test of the sensor included exposure to an analyte for 5 min followed by dry air purging for 10 min to restore the sensor. The gas standards used contained 2 ppm NO_2_ and 200 ppm NH_3_ in dry air, and CO (5.1 vol%)/CO_2_ (5 vol%)/H_2_ (5 vol%) in argon. To achieve different analyte concentrations, standard gas was mixed with synthetic air in the gas system using four mass flow controllers (Figure S1). Tests with 1 ppm NO_2_ at a relative humidity (RH) of 25%, 37.5%, and 50% were conducted by adjusting the flow rates of dry air, humid air with RH of 100%, and standard gas with three mass flow controllers. Air with a RH = 100% was obtained by passing dry air through a flask containing deionized water. The total gas flow rate in all measurements was 300 mL/min. The relative response of the sensor was calculated as: (I_g_ − I_0_)/I_0_ × 100%, where I_0_ and I_g_ are the current in the initial state (baseline) and the current in the presence of the analyte, respectively [49,50]. Response and recovery times were defined as the time required to achieve 90% of the full response to the analyte and 10% of the baseline when exposed to dry air, respectively.

### 2.3. DFT Calculations

The calculations were carried out using the projector-augmented-wave method implemented in the Quantum ESPRESSO package, version 7.0 [51], the Perdew–Burke–Ernzerhof function [52] in the generalized gradient approximation and Grimme D2 dispersion corrections [53]. The plane wave cutoff was 50 and 400 Ry for the kinetic energy and charge density, respectively. A 4 × 4 × 1 supercell was used for the hexagonal WS_2_ monolayer. The nanoribbon was four WS_2_ units wide. A 15 Å vacuum space separated the periodic copies in the vertical direction, which avoids interlayer interactions. The Brillouin zone integration was performed with a 3 × 3 × 1 and 3 × 1 × 1 Monkhorst–Pack grid for the monolayer and nanoribbon models, respectively. Atomic positions were optimized until the forces on all atoms were less than 0.045 eV/Å. Spin-polarized calculations were used to obtain the total energy. Adsorption energy was calculated as: E_ads_ = E_model_ − (E_WS2_ + E_mol_), where the terms are the total energies of WS_2_ with adsorbed molecule and isolated components. Bader analysis was performed to obtain atomic charges [54].

## 3. Results

### 3.1. Structure and Composition of WS_2_ Films

**Figure 1:**
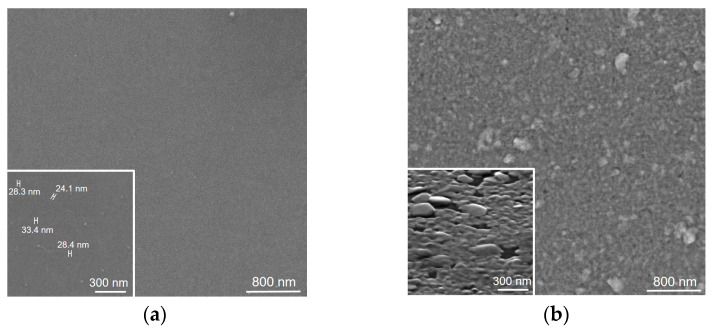
(**a**) SEM image of the surface of W layer sputtered on the SiO_2_/Si substrate for 20 s. The inset shows sizes measured for some nanoparticles. (**b**) SEM image of the surface of the WS_2_ film grown by sulfurization of the 20-s sputtered W layer. The inset shows the image recorded at a 70° angle to the film surface.

We first compared the changes in the structure of the metal layer before and after its interaction with sulfur vapor at 1000 °C. Figure 1a shows the SEM image of the tungsten surface formed on the SiO_2_/Si substrate as a result of metal sputtering for 20 s. The sputtering procedure used results in a uniform coating of the substrate with a homogeneous W layer and rare spherical nanoparticles on the surface. The size of these nanoparticles varies from ~24 to ~33 nm (inset in Figure 1a). After sulfurization, the morphology of the sample changes significantly (Figure 1b). The resulting film consists of densely packed nanoparticles ~70–120 nm in size. Quite large nanoparticles of up to ~300 nm in the in-plane size are most likely products of the sulfurization of spherical nanoparticles present on the surface of the sputtered tungsten layer. The SEM image taken at a 70° angle to the film surface shows that the smaller nanoparticles are horizontally oriented and in most cases merged with their neighbors, while the larger nanoparticles have a slightly tilted orientation (inset in Figure 1b).

**Figure 2:**
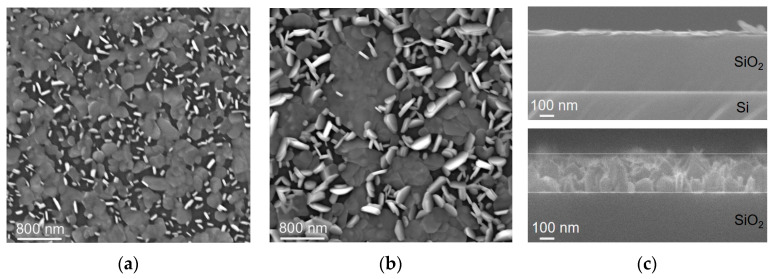
SEM images of the surface of (**a**) WS_2_-30s film and (**b**) WS_2_-90s film. (**c**) SEM cross-section images of WS_2_-30s (upper panel) and WS_2_-90s (bottom panel) grown on SiO_2_/Si substrates. The thin horizontal lines in the bottom panel were used to estimate film thickness.

Sulfurization of thicker W layers yields films consisting of vertical and horizontal WS_2_ nanoparticles (Figure 2). The edges of the vertical nanoparticles are visible as bright stripes in the SEM images. The nanoparticles, mostly aligned along the sample surface, have a cloudy contrast. According to the SEM data, both the thickness and the in-plane size of the nanoparticles are nearly twice as large in the film WS_2_-90s (Figure 2b) as in the film grown on a thinner W seed layer (Figure 2a). The ratio of vertical to horizontal nanoparticles in the films appears to be the same. The cross-section images of the WS_2_/SiO_2_/Si structures (Figure 2c) allowed us to estimate the WS_2_ film’s thickness, which is ~30 nm for WS_2_-30s and ~260 nm for WS_2_-90s. The image of the thicker film clearly shows vertically oriented sheets. To obtain cross-section images, the silicon substrate was broken. This mechanical action did not result in the separation of the WS_2_ film from the substrate, indicating a fairly good contact between them.

**Figure 3:**
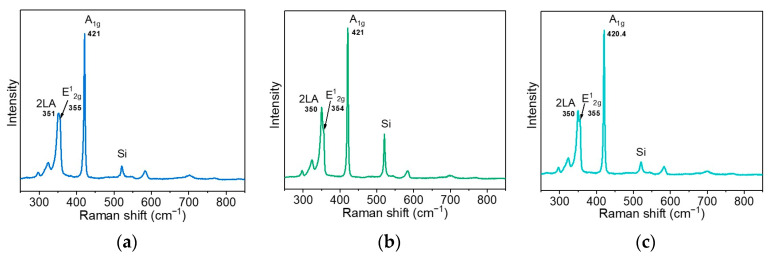
Raman spectra of (**a**) WS_2_-20s (**b**) WS_2_-30s, and (**c**) WS_2_-90s films measured at 514 nm.

The Raman spectra measured for WS_2_-20s, WS_2_-30s, and WS_2_-90s at 514 nm are compared in Figure 3. The spectra show two intense peaks: a narrow peak at 420.4–421 cm^−1^ corresponding to the out-of-plane A_1g_ mode and a broader peak at a lower Raman shift, which is the overlap of the in-plane E^1^_2g_ mode at 354–355 cm^−1^ and the 2LA mode at 350–351 cm^−1^. The peak positions are characteristic of multilayered 2H-WS_2_ [55]. The second-order 2LA mode is activated by defects, in particular sulfur vacancies [56]. No obvious differences were observed between the Raman spectrum of the WS_2_-20s film, where the particles have an almost horizontal orientation, and the spectra of the WS_2_-30s and WS_2_-90s films with mixed horizontal and vertical morphologies. This is consistent with the data reported previously for thin WS_2_ films prepared by sulfurizing electron-beam evaporated tungsten [57]. The spectra of our samples do not contain noticeable peaks in the region of 700–810 cm^−1^ from stretching vibrations of W–O bonds [58]. Therefore, the synthesis conditions used exclude the formation of oxidized tungsten nanoparticles.

**Figure 4:**
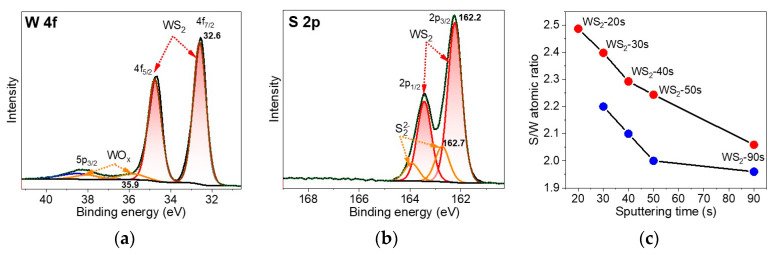
XPS (**a**) W 4f spectrum and (**b**) S 2p spectrum of WS_2_-30s film. (**c**) XPS-derived S to W atomic ratio for the WS_2_ films synthesized using W layers sputtered for various times (red dots—as prepared samples, blue dots—after annealing at 150 °C in argon).

The chemical states of the tungsten and sulfur in the WS_2_ films were elucidated by XPS analysis of the WS_2_-30s sample taken as an example. The W 4f spectrum showed an intense doublet with the energy of the spin-orbit 4f_7/2_ component at 32.6 eV (Figure 4a) corresponding to the W^4+^ state in 2H-WS_2_ [59,60]. A very weak doublet at a higher energy (W 4f_7/2_ at 35.9 eV) is attributed to the oxidized forms of tungsten. We propose the formation of W-terminated edges of the WS_2_ layers, which can attach to oxygen when exposed to laboratory air. The S 2p spectrum confirms the formation of the 2H-WS_2_ phase, since the binding energy of the 2p_3/2_ component of the main doublet is 162.2 eV (Figure 4b). The weaker doublet on the higher energy side (S 2p_3/2_ at 162.7 eV) corresponds to the S_2_^2−^ states [60] due to the dimerization of two sulfur atoms located at the edges of WS_2_, similar to what happens for MoS_2_ [61].

The S to W atomic ratio for the WS_2_ films synthesized using W layers sputtered for various durations was determined using survey XPS spectra (Figure S2a). The highest S/W atomic ratio (2.5) was observed for the WS_2_-20s sample, and it gradually decreases in the set of samples WS_2_-20s > WS_2_-30s > WS_2_-40s > WS_2_-50s > WS_2_-90s (Figure 4c). This behavior is associated with a decrease in the number of edge sulfur atoms S_2_^2−^ and, as a consequence, particle coarsening with increasing film thickness. Before testing, the sensors were annealed in argon at 150 °C for 40 min. XPS analysis showed that this annealing procedure led to a partial loss of sulfur (Figure S2b). As a result of the removal of weakly bound edge S_2_^2−^ groups, the stoichiometric ratio of the films approaches the exact stoichiometry (Figure 4c).

### 3.2. Sensor Properties

**Figure 5:**
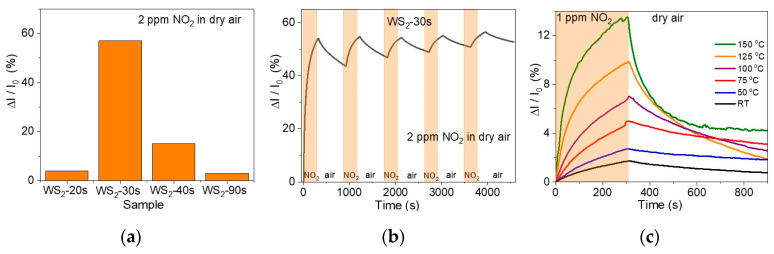
(**a**) Relative response of WS_2_ sensors when first exposed to 2 ppm NO_2_ in dry air at room temperature. (**b**) Relative response of the WS_2_-30s sensor to successive exposures of 2 ppm NO_2_ in dry air at room temperature. (**c**) Relative response of the WS_2_-30s sensor to 1 ppm NO_2_ at different operation temperatures.

Testing the sensor elements to 2 ppm NO_2_ in dry air at room temperature revealed a highest relative response of 57% for the WS_2_-30s film in the first cycle (Figure 5a). The presence of vertically oriented WS_2_ nanoparticles in this film significantly increases the response as compared to that of the WS_2_-20s sensor, which consists of horizontal nanoparticles (Figure 1b). The decrease in the sensor response in the series WS_2_-30s > WS_2_-40s > WS_2_-90s (Figure 5a) corresponds to the trend of increasing the thickness of the WS_2_ film.

After air purging, the WS_2_-30s sensor recovered by only 25% and gradually lost sensitivity in subsequent tests (Figure 5b). Its relative response decreased to 5.5% after the fifth analyte exposure, while the sensor recovery to the baseline increased to approximately 80%. The decrease in the sensor response during cycling may be due to the presence of high-energy adsorption sites on its surface. These sites are irreversibly occupied by adsorbates at room temperature, which may explain the improvement in sensor recovery over run-to-run tests.

To test the ability of NO_2_ molecules to escape from high-energy sensor sites, we performed measurements at elevated temperatures (Figure 5c and Figure S3). Figure 5c compares the changes in the relative response of WS_2_-30s upon adsorption of 1 ppm NO_2_ and subsequent air purging at room temperature and in the range from 50 to 150 °C with a step of 25 °C. As the temperature increases, the sensor response gradually rises, reaching a value of 13.5% at 150 °C, but the sensor recovery at this temperature is only 68%. The best recovery ~81% was observed at 125 °C, which can be considered optimal for NO_2_ desorption from the WS_2_-30s surface.

**Figure 6:**
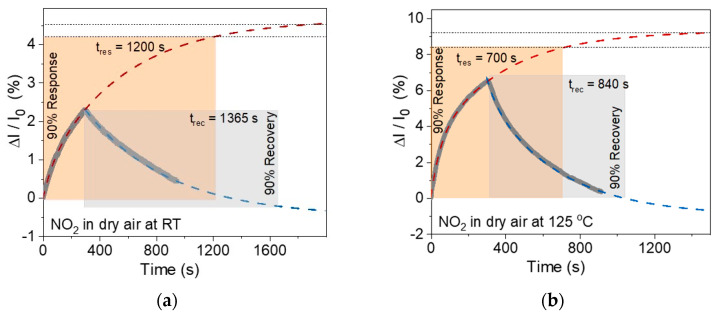
Response/recovery time determined for the WS_2_-30s sensor exposed to 1 ppm NO_2_ in dry air at (**a**) room temperature and (**b**) 125 °C. The dashed lines approximate the experimental curves to analyte saturation (red line approaching to the grey dotted line) and baseline (blue line).

Figure 5c shows that the WS_2_-30s sensor does not achieve equilibrium response and recovery under the test conditions used with 5 min of 1 ppm NO_2_ exposure and 10 min of dry air purge. To determine the response time and recovery time at room temperature and 125 °C, the recorded curves were fitted with lines corresponding to the saturation response for the analyte and the initial baseline (Figure 6), respectively. The response/recovery times are 1200/1365 s at room temperature (Figure 6a) and 700/840 s at 125 °C (Figure 6b). Upon reaching response saturation, the relative response of the sensor increases by 1.8 times at room temperature and 1.3 times at 125 °C as compared to the values obtained in tests using 5-min analyte exposure (Figure 5c).

**Figure 7:**
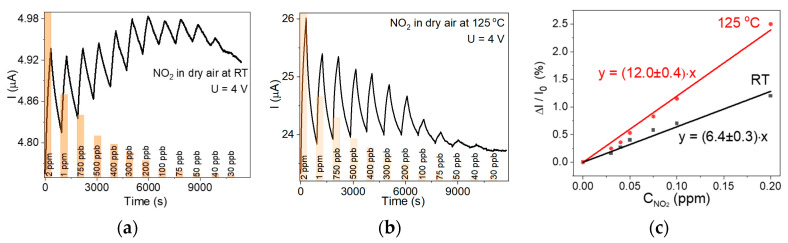
Change in WS_2_-30s sensor current as a function of NO_2_ concentration in dry air (**a**) at room temperature (RT) and (**b**) at 125 °C. The voltage is 4 V. (**c**) Response dependence on NO_2_ concentration obtained for the WS_2_-30s sensor at room temperature (RT) and 125 °C.

Figure 7a,b shows the change in the WS_2_-30s sensor current depending on the NO_2_ concentration in dry air. At room temperature, the baseline has a significant drift (Figure 7a), which is almost completely eliminated at 125 °C (Figure 7b). The sensor reliably detects 30 ppb NO_2_ at both operation temperatures, but the response becomes more intense at 125 °C. The concentration of 30 ppb is the limit defined by the NO_2_ gas standard and the equipment used, so the detection limit of the sensor is below this value. The experimental curves presented in Figure 7a,b were used to plot the relative response of the WS_2_-30s sensor against NO_2_ concentration. The data obtained for NO_2_ concentrations down to 0.2 ppm were fitted with straight lines (Figure 7c). The LOD of the sensor was evaluated from the slope of the lines, taking into account a signal-to-noise ratio of 3. The obtained LOD value is 15 ppb at room temperature and decreases to 8 ppb at 125 °C.

The relative response and LOD obtained for our most sensitive sensor WS_2_-30s are compared with the data found in the literature for resistive sensors fabricated solely from WS_2_ (Table 2). Most of the WS_2_ sensors can detect NO_2_ concentrations at the ppb level at room temperature. The best results were obtained for samples grown on SiO_2_/Si substrates using CVD [29,38,42] or pulsed laser deposition [44]. These methods allowed the WS_2_ nanoparticles to be assembled into a film with an extended surface area. Although the nanoparticles varied in morphology, they all had abundant exposed edges. The above structural characteristics are also realized in our WS_2_-30s sensor, which showed a very low theoretical LOD value, especially at a moderate temperature of 125 °C.

**Figure 8:**
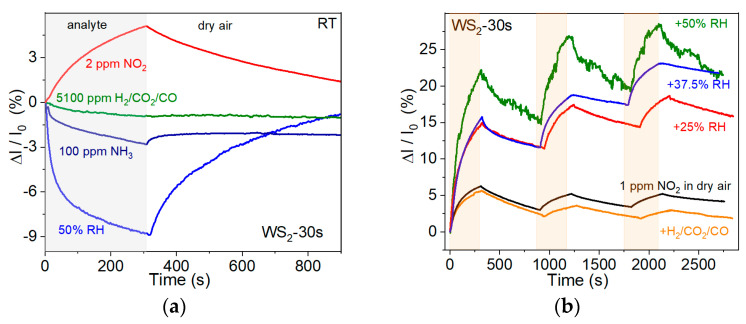
(**a**) Selectivity of WS_2_-30s sensor to 2 ppm NO_2_, gaseous mixture 5100 ppm H_2_/5100 ppm CO_2_/5100 ppm CO, 100 ppm NH_3_, and 50% RH at room temperature. (**b**) Comparison of the dynamic performance of WS_2_-30s at room temperature for 1 ppm NO_2_ in dry air, 1 ppm NO_2_ in humid air with different RH values, and 1 ppm NO_2_ in dry air containing 5100 ppm each of H_2_, CO_2_, and CO. Orange and white vertical strips correspond to the sensor exposure to analyte and dray air, respectively.

The WS_2_-30s sensor showed very similar response values in the low NO_2_ concentration range of 30–50 ppb when operating at room temperature and 125 °C (Figure 7c). Since these concentrations need to be detected and low-power sensors are in high demand, the selectivity test was performed at room temperature. Figure 8a compares the relative response of the WS_2_-30s sensor to 2 ppm NO_2_, 100 ppm NH_3_, 50% H_2_O, and 5100 ppm CO_2_/H_2_/CO in air. The positive response value, corresponding to the increase in current when the sensor is exposed to oxidizing NO_2_ molecules, indicates the p-type conductivity of the WS_2_-30s film. The relative response values to the other analytes used are negative. The decrease in sensor current suggests that these molecules act as electron donors. The significantly lower relative response to the 100 ppm NH_3_ (~2.8%) and H_2_/CO/CO_2_ gas mixture (~0.9%) indicates that the WS_2_-30s sensor is selective for NO_2_.

Our sensor showed a high response (~9%) under the action of wet air with a RH value of 50% (Figure 8a). Since analyte detection under ambient conditions is an important task, we examined the WS_2_-30s sensor for 1 ppm NO_2_ in air with RHs of 25%, 37.5%, and 50% at room temperature. Cycling the sensor showed its repeatable response over at least three cycles (Figure 8b). The addition of H_2_, CO_2_, and CO (5100 ppm each) to NO_2_ had little effect on the sensor response. The concentration of these gases in air is usually lower than in the test conducted. Therefore, the sensor can be used to detect NO_2_ under practical conditions.

## 4. Discussion

**Figure 9:**
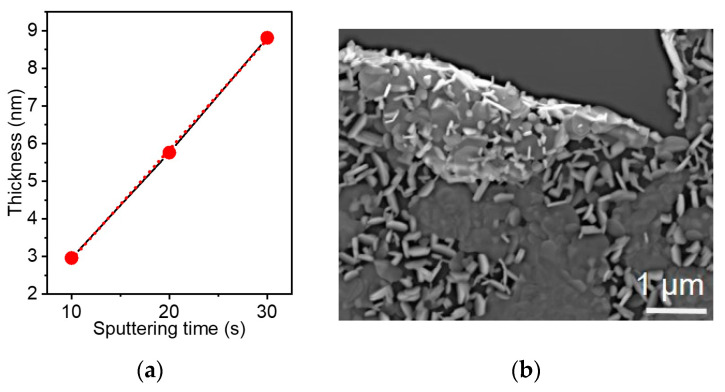
(**a**) W layer thickness on SiO_2_/Si substrate versus sputtering time. The red line approximates the black line connecting the experimental dots. (**b**) SEM image of WS_2_-90s showing the structure on both sides of the film.

In this work, WS_2_ films were grown by CVD sulfurization of W layers deposited on SiO_2_/Si substrates using a magnetron sputtering system. The sputtering time was varied from 10 to 90 s. The W layer thickness was estimated from the intensities of the XPS W 4f and Si 2p lines (Figure S4) using the Thickogram method [64]. This was done for samples sputtered for 10 s, 20 s, and 30 s, where the Si 2p line was visible in the spectra. The line y = 0.293x with the determination coefficient R^2^ equal to 0.999 approximates the obtained values (Figure 9a), so this dependence can be used to determine the thickness of longer sputtered layers.

Sulfurization of W layers less than 6 nm thick (20 s of sputtering) produces horizontally laid WS_2_ nanoparticles (inset in Figure 1b). The use of thicker W layers leads to the formation of horizontally and vertically oriented WS_2_ nanoparticles (Figure 2a,b). Similar morphological changes depending on the thickness of the W seed layer were observed for WS_2_ films synthesized using sulfur vapor at 700 °C [46]. The formation of vertical layers is explained by strains occurring in the metal fixed to the substrate as a result of its volume expansion during sulfurization [45].

The tungsten layer used to synthesize the WS_2_-30s film had a thickness of ~9 nm (Figure 9a). According to the SEM data, sulfurization of this layer led to an increase in the sample thickness to ~30 nm (Figure 2c). Since the in-plane size of WS_2_ nanoparticles in this film varies from ~70 to ~250 nm, we conclude that they are not perfectly vertical but tilted relative to the substrate surface.

Resistance measurements using the two-probe method showed the highest value for the WS_2_-30s film (~1.9 MΩ). The resistance of other films was several tens of kilo-Ohms. Conduction paths in the films are provided by contacting horizontal WS_2_ nanoparticles. These nanoparticles dominate in the WS_2_-20s film and are separated by inclined nanoparticles in the WS_2_-30s film. With the increase of the thickness of the sulfurizing W layer, the nanoparticles grow in size and merge. An SEM image of the WS_2_-90s film on the top and bottom sides shows that the horizontal nanoparticles are evenly distributed throughout the film depth and that they are larger than the vertical and inclined nanoparticles (Figure 9b). In this particular case, the adhesion of the film to the substrate was weaker, which allowed it to be separated. The fact that the detached film retained its integrity indicates that the WS_2_ nanoparticles are sufficiently well bonded to each other.

Comparison of the Raman spectra of WS_2_-20s, WS_2_-30s, and WS_2_-90s confirms the formation of smaller nanoparticles in the WS_2_-30s film, since the disorder-activated 2LA mode has the highest intensity relative to the in-plane vibration E^1^_2g_ mode for this sample (Figure 3). The appearance of the 2LA mode may be related to sulfur vacancies present in the WS_2_ lattice [56]. However, the CVD sulfurization of all samples was carried out under identical conditions. Therefore, we do not expect a significant difference between them in the number of lattice defects as well as in the oxidized states of sulfur and tungsten.

**Figure 10:**
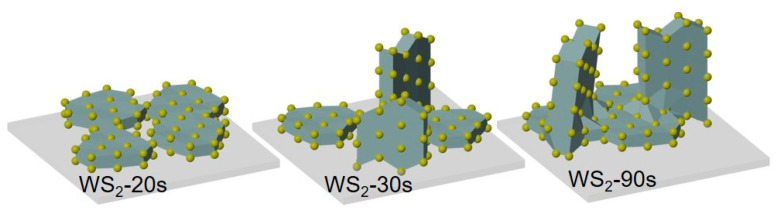
Schematic representation of the evolution of the WS_2_ film morphology depending on the sputtering time of the W layer onto the SiO_2_/Si substrate (shown in grey). The tungsten planes are colored in green-grey, the yellowish balls are sulfur atoms.

The morphology changes of the WS_2_-20s, WS_2_-30s, and WS_2_-90s films are shown schematically in Figure 10. The highest sensor response of the WS_2_-30s film (Figure 5a) can be attributed to its large surface area provided by (1) the nanometer film thickness, (2) the small size of its constituent nanoparticles, and (3) the presence of many vertical and tilted nanoparticles. The highest resistivity of this film among the studied samples could be explained by the weaker contacts between horizontal and vertical WS_2_ nanoparticles (central model in Figure 10). The adsorption of the analyte on the high-resistance material causes a strong change in the electric current, which is an additional factor contributing to the highest sensitivity of the WS_2_-30s film to NO_2_. It should be noted that the differences in the structure of the WS_2_ films, resulting in significantly different responses to NO_2_ adsorption, were achieved by changing only one parameter in the synthesis procedure, namely the time of the W layer sputtering on the substrate. To evaluate the reproducibility of the sensor obtained by this method, we repeated the synthesis and testing of the WS_2_-30s film. The sensors produced in two independent syntheses exhibited similar kinetic behavior and baseline drift when exposed to 2 ppm NO_2_/dry air at room temperature four times (Figure S5). Deviations in the absolute values of the sensor characteristics, such as relative response and recovery, can be minimized by more precise control of all synthesis parameters (substrate surface, sputtering duration of tungsten, synthesis temperature and gas flow rate during the CVD step, etc.).

**Figure 11:**
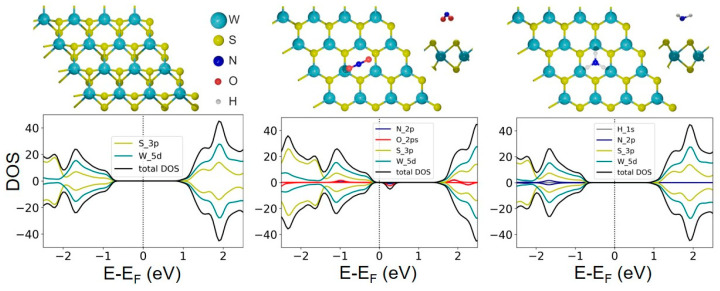
Calculated supercells (**top**) and density of states (DOS, **bottom**) for a hexagonal WS_2_ monolayer (**left panel**), monolayer with adsorbed NO_2_ (**center panel**), and monolayer with adsorbed NH_3_ (**right panel**). Contributions of orbitals of different elements are separated in the total DOS. The Fermi energy is taken to be zero.

The conductivity of the WS_2_-30s sensor increased when exposed to NO_2_ and decreased under the influence of NH_3_ (Figure 8a). The relative response was significantly smaller in the second case. To study the interaction of WS_2_ with these molecules, we employed DFT. The calculation of a hexagonal WS_2_ monolayer determined the band gap to be 1.71 eV (left in Figure 11). The 5d orbitals of tungsten dominate the density of states (DOS) near the top of the valence band, while they are strongly hybridized with the 3p orbitals of sulfur at the bottom of the conduction band. The NO_2_ molecule is oriented with its oxygen atoms toward the basal WS_2_ plane with a shorter O–S distance of 3.07 Å (center in Figure 11). The lowest energy position of the NH_3_ molecule is due to the orientation of the N atom to the hole of the hexagon at a distance of 3.28 Å from the sulfur atoms (right in Figure 11). The calculated adsorption energies E_ads_ are −0.17 eV for NO_2_ and −0.19 eV for NH_3_. The negative energy indicates a gain from adsorption. The very close E_ads_ values suggest that NO_2_ and NH_3_ compete for the WS_2_ surface.

The charges induced on the NO_2_ and NH_3_ molecules adsorbed on the WS_2_ monolayer are −0.09*e* and +0.03*e*, respectively. The opposite signs of the charges correlate to the increase/decrease of the WS_2_-30s sensor current during NO_2_/NH_3_ adsorption (Figure 8a). The interaction of NO_2_ with WS_2_ leads to the appearance of impurity empty states near the valence band of the monolayer (center in Figure 11). These states correspond to the lowest unoccupied molecular orbital of NO_2_. The orbitals of the adsorbed NH_3_ are located below −1.5 eV (right in Figure 11), so they are mixed with the orbitals of WS_2_. Our results on the different types of interaction of WS_2_ with NO_2_ and NH_3_ are consistent with other theoretical and experimental data [34,41,65]. Based on the DFT calculations, the higher response of the WS_2_ sensor to NO_2_ as compared to NH_3_ is related to (1) larger transferred charge and (2) easier electron transport due to the impurity states in the band gap.

In addition to the DFT-based explanation for the selectivity of WS_2_ to NO_2_, we should also consider the experimental data on the electronic state of the WS_2_ film under study. The direction of the current change during adsorption of the electron acceptor NO_2_ and the electron donor NH_3_ (Figure 8a) indicates p-type doping of the sensor material. This type of doping is due to the presence of WO_x_ states [23] and excess sulfur [66] as shown by the analysis of XPS spectra (Figure 4a,b). The holes generated as a result of NO_2_ adsorption greatly affect the conductivity of the p-doped sensor.

When first exposed to 2 ppm NO_2_ in dry air, the WS_2_-30s sensor showed a 57% relative response and only a 25% recovery at room temperature (Figure 5b). The DFT calculated adsorption energy of NO_2_ on the WS_2_ surface is −0.17 eV, and this small value cannot account for the observed poor recovery of the sensor. The high-energy regions may correspond to the edges of WS_2_.

**Figure 12:**
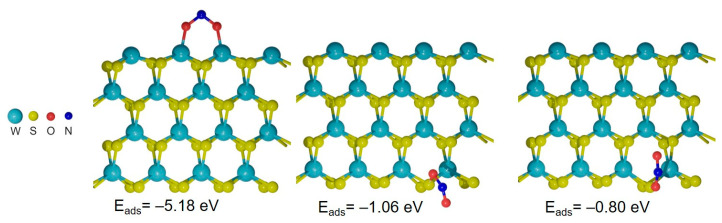
Optimized models of WS_2_ nanoribbons with NO_2_ located at the W-edge (**left panel**) and S-edge (**central** and **right panels**).

To study the interaction of NO_2_ with the WS_2_ edges, we used a zigzag nanoribbon with W- and S-edges on opposite sides (Figure 12). The NO_2_ molecule is attached by both oxygen atoms to the W-edge (left model in Figure 12). The O–W bond length is 1.89 Å and the adsorption energy is −5.18 eV. The high E_ads_ value and short bonds indicate chemical adsorption of the molecule. Calculation results show that if the WS_2_ sensor contains W-edges, these edges should be rapidly occupied by NO_2_ molecules (and possibly other oxygen-containing molecules present in the surrounding atmosphere). Removal of these tightly attached NO_2_ molecules requires temperatures that are too high for the sensor to operate. The S-edges also attract NO_2_. According to calculations, the E_ads_ value for the NO_2_ molecule in the model shown in the center of Figure 12 is −1.06 eV, which is significantly weaker than in the case of the W-edge. An even lower E_ads_ is found when a molecule located near the S-edge interacts with two sulfur atoms (right model in Figure 12). Molecules from such positions can be detached when the sample is heated. The charge of the NO_2_ molecule in the right model in Figure 12 is −0.43*e*, which is more than four times the charge of the molecule adsorbed on the basal plane. Therefore, such molecules could provide a larger change in the conductivity of the WS_2_ layer and, as a result, a large response of the sensor, which was observed in tests performed at elevated temperatures (Figure 5c).

**Figure 13:**
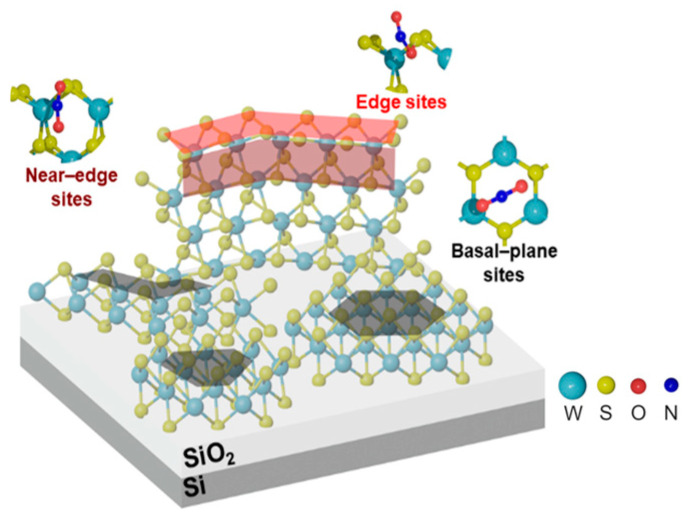
Schematic structure of the WS_2_ gas sensor with the preferred sites for NO_2_ adsorption. The red, brown and black areas of the sensor indicate high-, medium, and low-energy NO_2_ adsorption sites, respectively.

Based on the results of DFT calculations, the following structure of a thin-film sensor, consisting of WS_2_ layers oriented vertically and horizontally along the substrate, can be proposed (Figure 13). The horizontal layers provide mainly a basal plane for adsorption. NO_2_ molecules interact weakly with the plane, resulting in a relatively small sensor response but easy recovery. The vertical layers have additional adsorption sites, such as protruding edges. The W-edges are usually oxidized if the sample has been in contact with air atmosphere and should not interact with NO_2_. The interaction of NO_2_ molecules with the S-edges is strong, and this explains the poor recovery of the sensor at room temperature. However, the sites located near the WS_2_ edges are optimal for NO_2_ adsorption. This adsorption is accompanied by a large charge transfer and does not have a high energy. According to SEM data (Figure 2a,b), the nanoparticles comprising the WS_2_ films are dominated by basal-plane adsorption sites and therefore contribute significantly to the overall reproducible response of the sensor, especially at room temperature.

The WS_2_-30s film consists of vertical and horizontal nanoparticles whose dimensions are smaller than those of nanoparticles produced by the sulfurization of thicker W layers. The smaller in-plane size provides a higher proportion of near-edge sites relative to basal-plane sites, making this sensor highly sensitive to NO_2_ (Table 2). However, the sensor suffers from long response/recovery times. The long response and recovery times of the WS_2_-30s sensor are due to the presence of multiple adsorption sites of different energies on its surface. A further direction for developing WS_2_-based sensors synthesized by this method is obtaining films with an optimal nanoparticle size. The time characteristics can also be improved by depositing semiconductor nanoparticles such as metal oxides [32,33] and metal sulfides [67] on the surface of the sensor material. WS_2_ films grown on the appropriate substrate are well suited for such modifications.

## 5. Conclusions

Thin films of WS_2_ were grown on SiO_2_/Si substrates by sulfurizing tungsten layers at 1000 °C in the presence of H_2_. The thickness of the tungsten layer was estimated by analyzing the XPS spectra of samples obtained by magnetron sputtering for 10–30 s. Sulfurizing W layers with a thickness of less than about 6 nm resulted in the formation of hexagonal nanoparticles aligned along the substrate. Then, the morphology of the films changed radically to co-existence of the horizontal nanoparticles with the vertical ones. According to the SEM study of the films obtained using W layers sputtered for 30 and 90 s, the thickness of the WS_2_ coating on the SiO_2_/Si substrate is ~30 and 260 nm, respectively. In the film WS_2_-90s, the nanoparticle sizes were twice as large as those in the film WS_2_-30s. Raman and XPS spectra measured for the films showed peaks characteristic of 2H-WS_2_. The films were tested as resistive sensors for NO_2_ detection in air atmosphere. The greater sensitivity was observed for WS_2_-30s and attributed to the nanometer film thickness, the developed surface due to the presence of vertical nanoparticles, and the high resistance of the film caused by the growth of vertical nanoparticles between horizontal ones. The WS_2_-30s sensor showed a significantly higher relative response to NO_2_ as compared to NH_3_. Based on DFT calculations, this was attributed to the appearance of impurity states in the band gap and a higher charge transfer during NO_2_ adsorption. For p-doped WS_2_, as in our case, the latter leads to a significant increase in the sample conductivity. Testing of the WS_2_-30s sensor revealed a baseline drift with repeated exposure to NO_2_ and dry air at room temperature, which became negligible at 125 °C. According to DFT calculations, high-energy NO_2_ adsorption occurs near the edges of the WS_2_ nanoparticles, and molecules at the S-edges can be detached upon heating. The possibility of practical applications of the synthesized WS_2_ films as gas sensors was demonstrated by measurements in humid air and in the presence of interfering gases H_2_/CO_2_/CO.

## Figures and Tables

**Table 1:** Operation temperature (RT—room temperature), limit of detection (LOD), response/recovery time (τ_res_/τ_rec_) and gases in selectivity test (ST) reported for highly sensitive NO_2_ sensors.

Material	Temperature	LOD	τ_res_/τ_rec_ (s)	Gases in ST	Reference
CuO	100 °C	<300 ppb	64/274 at 5 ppm	NH_3_, CO, CO_2_, H_2_S	[18]
In_2_O_3_ nanosheets	50 °C	5 ppb	690/1951 at 2 ppm	NH_3_, CO_2_, H_2_S	[14]
NiO/N-rGO	100 °C	<1 ppb	660/2700 at 0.8 ppm	NH_3_, CO_2_	[19]
N-rGO	100 °C	39 ppb	600/2640 at 0.8 ppm	NH_3_, CO_2_	[19]
SWCNTs	150 °C	12 ppb	208/193 at 1 ppm	not studied	[20]
MoS_2_ exfoliated	RT	100 ppb	600/1200 at 1 ppm	NH_3_, CH_4_, SO_2_	[21]
WS_2_/rGO	RT	363 ppb	58/627 at 10 ppm	NH_3_, CO_2_, H_2_S, SO_2_, N_2_O	[22]
WS_2_@Carbon fibers	RT	31 ppb	54/305 at 10 ppm	NH_3_, CO, CO_2_, H_2_S, SO_2_, H_2_	[16]
MoS_2_/Graphene	RT	18 ppb	254/1262 at 1 ppm	NH_3_, CO, CO_2_, H_2_S, H_2_	[23]
N-Carbon dots/SnS_2_	RT	10 ppb	9/132 at 1 ppm	NH_3_, CO, CO_2_, SO_2_, H_2_	[24]
V_2_CT_x_/SnS_2_	RT	300 ppb	4.8/4.7 at 5 ppm	NH_3_, CO_2_, H_2_S, CH_4_, NO	[25]
Mo_2_TiC_2_T_x_/MoS_2_	RT	<3 ppb	61/154 at 10 ppm	NH_3_, CO_2_, H_2_S, CH_4_, NO	[26]
MoSe_2_-WS_2_@Si	RT	<50 ppb	69/66 at 50 ppb	NH_3_, CO, H_2_S	[27]

**Table 2:** Response of resistive sensors made from WS_2_ solely to NO_2_ gas and LOD determined at room temperature.

Material	Concentration	Response (%)	LOD	Reference
multilayered WS_2_	50 ppb	17.5	<20 ppb	[29]
four-layered WS_2_	25 ppm	8.7	<25 ppm	[31]
WS_2_ nanotriangles	200 ppb	4.0	N/A	[32]
two layered WS_2_	1 ppm	419	N/A	[36]
WS_2_ nanoflakes	300 ppb	0.5	N/A	[38]
	800 ppb	26.6	~50 ppb (150 °C) ^1^	
WS_2_ nanosheets	10 ppm	1.13	N/A	[15]
WS_2_ nanorods	5 ppm	151.2	13.7 ppb (theor) ^2^	[42]
vertical WS_2_ flakes	50 ppb	4	<50 ppb (40 °C) ^1^	[44]
WS_2_ nanosheets	100 ppm	14	<50 ppb	[62]
WS_2_ nanosheets	2 ppm	8	3.16 ppm (theor) ^2^	[63]
WS_2_ nanosheets	2 ppm	7	523 ppb (theor) ^2^	[22]
thin WS_2_ film	40 ppb	0.27	15 ppb(theor) ^2^	this work
	40 ppb	0.36	8 ppb (theor ^2^, 125 °C) ^1^	this work

^1^ Temperature of sensor operation. ^2^ Theoretical value of LOD.

## Data Availability

The data that support the findings of this study are available on request from the corresponding author.

## Supplementary Materials

The following supporting information can be downloaded at: https://www.mdpi.com/article/10.3390/nano15080594/s1, Figure S1: Schematic illustration of the laboratory setup for gas sensor measurements. Figure S2: (a) XPS survey spectra measured for WS_2_ films grown on SiO_2_/Si substrates by sulfurization of W layers sputtered for 20 s, 30 s, 40 s, 50 s, and 90 s. (b) XPS survey spectra measured for the annealed WS_2_ films before their test as sensors. Figure S3: Change in WS_2_-30s sensor current when exposed to 1 ppm NO_2_ in dry air at different temperatures. Figure S4: XPS Si 2p spectra and W 4f spectra measured for W layers deposited on SiO_2_/Si substrates during 10 s, 20 s, 30 s, and 40 s of magnetron sputtering. Figure S5: Comparison of relative response plots of two WS_2_-30s films produced in independent syntheses for four cycles with 2 ppm NO_2_ in dry air at room temperature.
