# Supplementary material for: Thin Films of Tungsten Disulfide Grown by Sulfurization of Sputtered Metal for Ultra-Low Detection of Nitrogen Dioxide Gas
Source: Nanomaterials (Basel). 2025 Apr 12;15(8):594. doi: 10.3390/nano15080594 (PMC12029938; doi:10.3390/nano15080594)
Supplement: Supplementary file 1 [file nanomaterials-15-00594-s001.zip › nanomaterials-3530347-supplementary.pdf]

Supplementary Materials

Thin Films of Tungsten Disulfide Grown by Sulfurization of Sputtered Metal for Ultra-Low Detection of Nitrogen Dioxide Gas

Anastasiya D. Fedorenko, Svetlana A. Lavrukhina, Victor A. Alekseev, Vitalii I. Sysoev, Veronika S. Sulyaeva, Alexander V. Okotrub, and Lyubov G. Bulusheva*

Nikolaev Institute of Inorganic Chemistry SB RAS, 3 Acad. Lavrentiev Ave., 630090 Novosibirsk, Russia

*Correspondence: bul@niic.nsc.ru (L.G.B.)

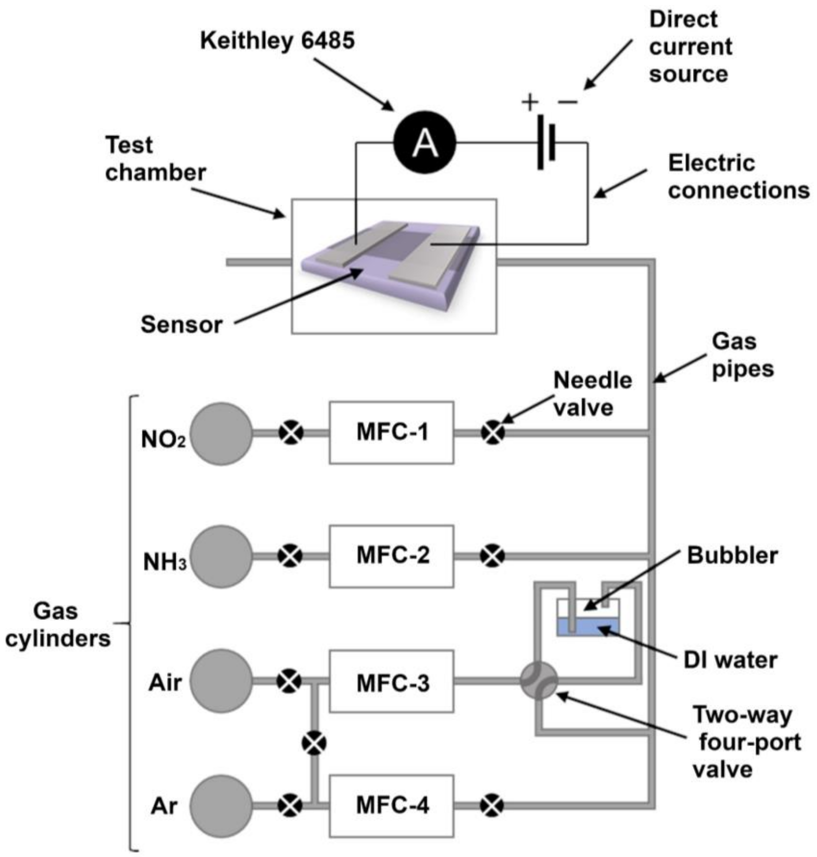

Figure S1: Schematic illustration of the laboratory setup for gas sensor measurements. “MFC” stands for Mass Flow Controller.

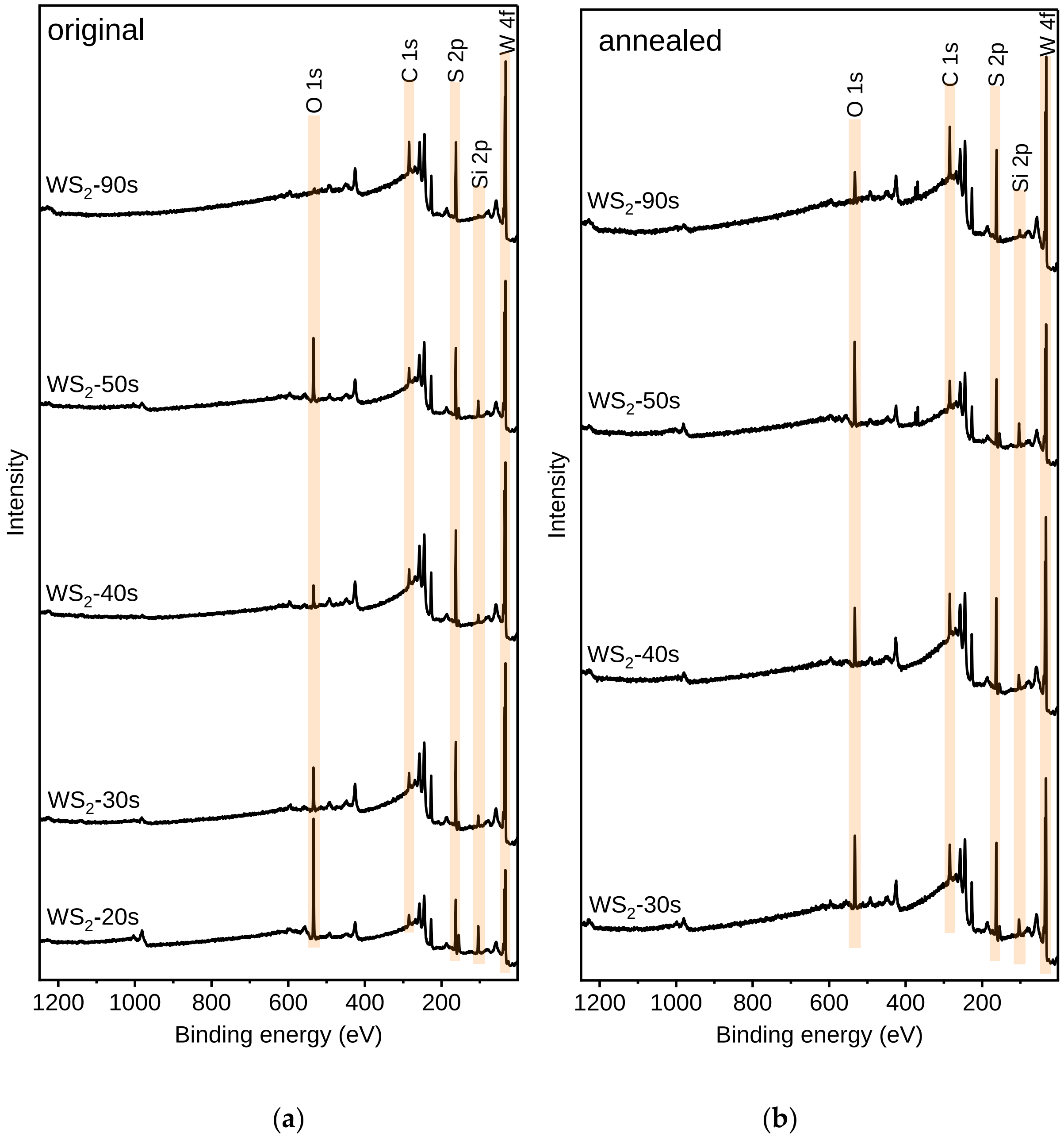

Figure S2: (a) XPS survey spectra measured for WS_2 films grown on SiO_2/Si substrates by sulfurization of W layers sputtered for 20 s, 30 s, 40 s, 50 s, and 90 s. **(b)** XPS survey spectra measured for the annealed WS_2 films before their test as sensors.

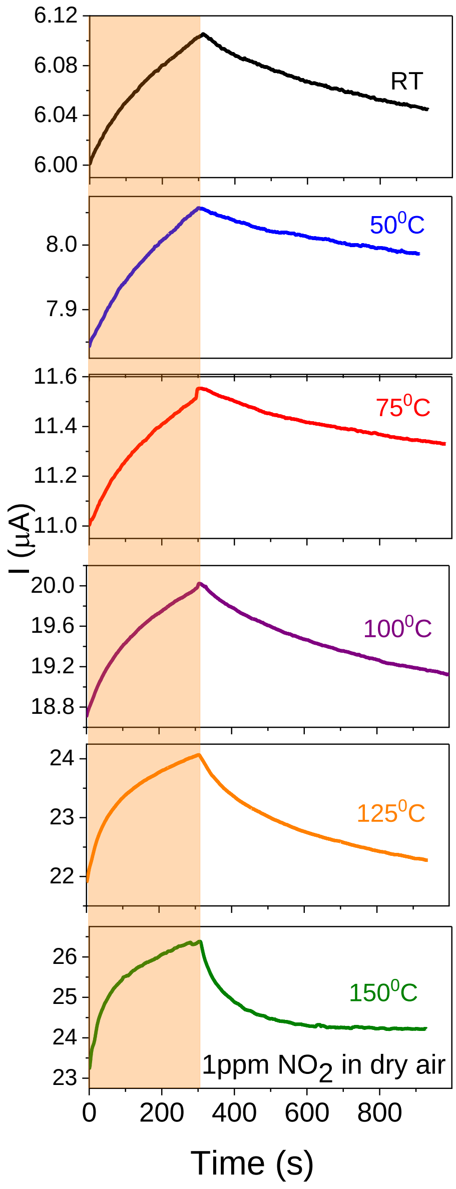

Figure S3: Change in WS_2-30s sensor current when exposed to 1 ppm NO_2 in dry air at different temperatures. The voltage is 4 V.

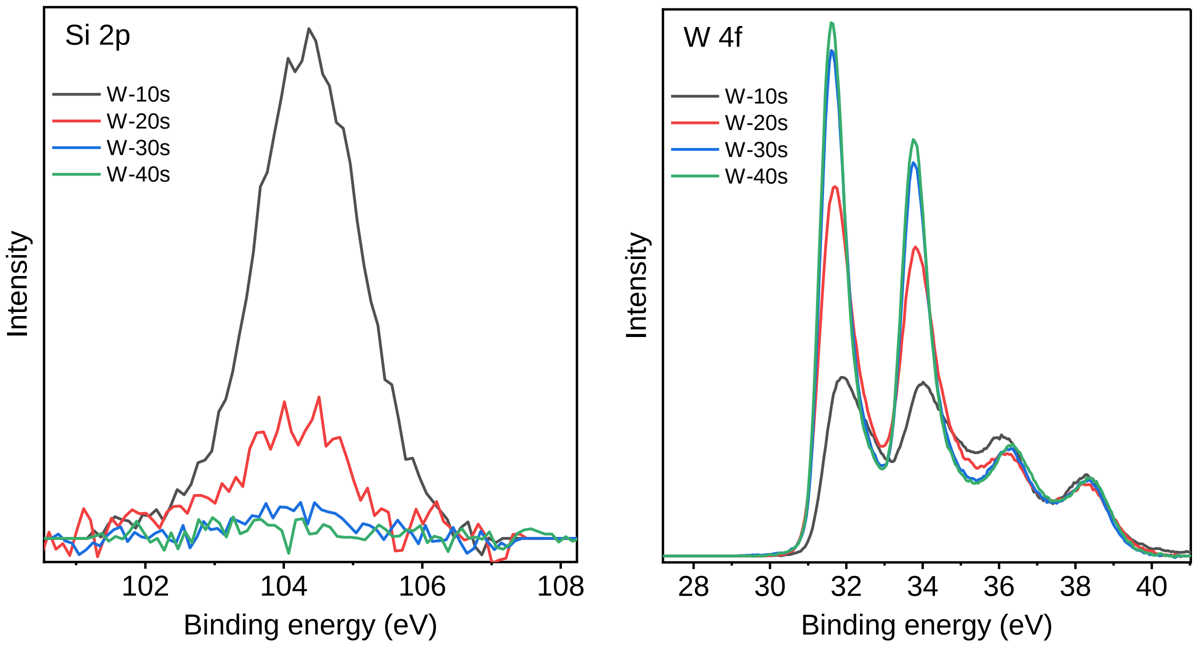

Figure S4: XPS Si 2p spectra and W 4f spectra measured for W layers deposited on SiO_2/Si substrates during 10 s, 20 s, 30 s, and 40 s of magnetron sputtering.

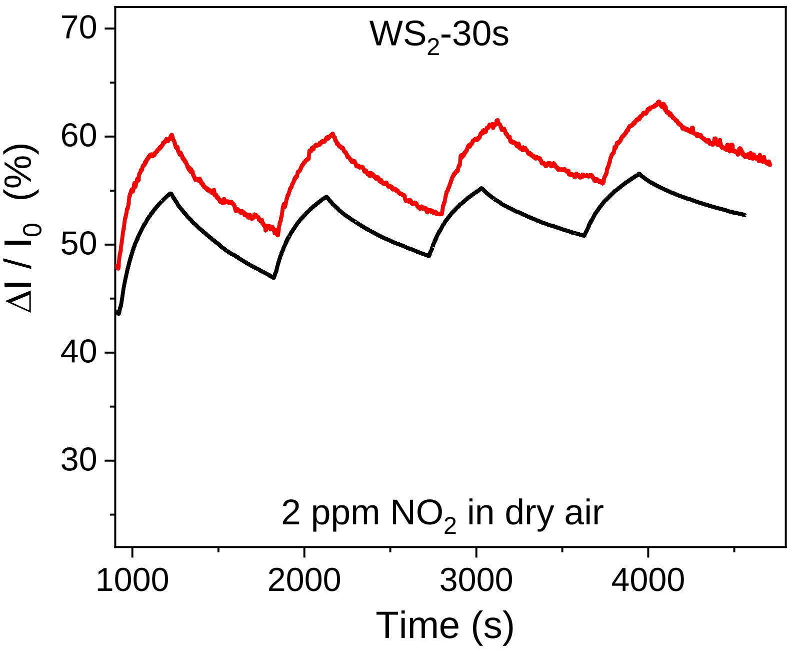

Figure S5: Comparison of relative response plots of two WS_2-30s films produced in independent syntheses for four cycles with 2 ppm NO_2 in dry air at room temperature.
